# News and Notes

**Published:** 1911-05

**Authors:** 


### NEWS *and* NOTES

### THE FIGHT AGAINST HOOKWORM DISEASE.

Dr. A. G. Fort, director of field sanitation in the fight being waged against the hookworm in the state of Georgia, has announced the following appointments to office in this field which have been indorsed by the state board of health and confirmed by the commission: Dr. C. E. Patillo is apponted to conduct examnations in the laboratory of hookworm specimens. Dr. A. W. Wood, at present in charge of the Wesley Memorial hospital, is appointed to the office of sanitary inspector to succeed Dr. W. C. Thompson, resigned.

Dr. B. Randall, of Atlanta, is appointed sanitary inspector to take the place of Dr. C. E. Pattillo, who has been removed to the laboratory. Dr. C. R. Henry, of Rome, Ga., is appointed inspector to fill the vacancy caused by th exepiration of the term of Dr. P. H. Fitzgerald, of Omaha, Ga., on May 15.

Dr. C. B. Greer, of Atlanta, is appointed to the office of sanitary inspector from the date of June 1, 1911. Dr. Carl C. Verner, of Toccoa, is appointed to conduct inspection work during the months of May, June, July and August.

Director Fort has outlined a vigorous campaign against the hookworm for the present season similar to the steps which will be taken in the other southern states under the supervision of the hookworm commission.

---

### MODERN HOSPITAL IN LARGEST HOTEL IN THE WORLD.

One of the numerous unique features to be offered by the new McAlpin Hotel, now in course of erection on the southwest corner of 34th Street and Broadway, New York City, is a fully equipped miniature hospital where cases, no matter how serious, can be treated with exactly the same care as in the best up-to-date private sanitarium. It is to be arranged so as to be able to comfortably accommodate twelve patients at one time. Expert surgeons, physicians and trained nurses will be in attendance so that surgical operations of any character can be skillfully handled at a few moments' notice.

This practical and extraordinary addition to hotel accommodations is to be situated on the 23rd floor of this largest hotel in the world so that a patient can enjoy the same quiet and comfort as though being treated in the most tranquil locality in spite of the fact that the McAlpin is to be the most centrally located hotel in New York City.

Expert surgeons and medical men have been consulted by Mr. Frank Andrews, the architect of the hotels, and plans are being made for this miniature hospital that it will be fitted with every modern appliance known to surgery in exactly the same manner as the best equipped hospital in any part of the country.

---

### REGULAR MEETING FULTON COUNTY MEDICAL SOCIETY, MACRH 2, 1911.

Dr. Theodore Toepel read a paper on "Treatment of Anterio Polio Myelitis."

Dr. Paullin said that this disease was on the increase, especially in the North and that its infectiousness was daily growing more evident. We have not yet isolated the germ of the disease and, therefore, the treatment is not specific. Emperically, it consists of the application of electricity and massage, physiologic rest in bed and passive motion. The operative question comes up later. Counter irritation is worthless and harmful. As the patient improves there is a return of Furadic sensibility.

Dr. Block said that he was glad that Dr. Toepel did not encourage the use of braces early in the disease, as they interfere with returning strength. Electricity should be used at least two years before braces or other surgical matter is applied. The tibialis anticus and the peroneii are likely to remain paralyzed throughout life. Rection of degeneration shows this. When after long treatment, the muscles do not respond, surgical intervention is indicated.

Dr. Toepel closed by saying that orthopedic measures are always harmonious with other treatment and tend to prevent the waste of the muscles as is indicated in the paper published elsewhere.

Dr. F. P. Calhoun read a paper on "Uncinariasis as a Cause of Cataract."

Dr. Daly said that the early age at which the cataract appeared in the majority of cases reported by Dr. Calhoun showed that there must be other than the ordinary cause for the disease and the finding of hook-worm in the bowels of the patient gave strong presumptive evidence that this gave rise to the toxemia, causing the cataract. Whether or not signs of invasion of the eye were to be found in all cases of hook-worm seemed to be a matter for thoughtful attention and investigation.

Dr. Block asked if Dr. Calhoun failed to find the hookworm in any of the cases which gave the symptoms he described. Dr. Calhoun said that they were always found in these cases. As to preliminary infection, an incipient cataract in Uncinariasis might possibly be stopped.

Dr. J. N. Brawner read a paper on "Epileptic Insanities."

Dr. Block said that the medica-legal aspects of epilepsy were important and difficult to decide upon. Amnesia of apparently purposive acts is frequent. He told of a case of a young man going from Atlanta to Newnan without knowing where he was or how he got there. This man might have committed a crime and would not have been responsible though he appeared to be so. Another case who had an attack the day before was taken into a dark room for examination of the eye. Whilst there he undressed and made a variety of queer motions and upon request replaced his clothes. A little later he was surprised to find his clothes were disarranged but had absolutely no recollection of his previous acts. Dr. Block would not agree that epileptics become great. Mahomet Napoleon and Caesar had few convulsions, if they were epileptics, and for that matter were always causing disturbances any way. The treatment of epilepsv has shown little or no advances lately.

Dr. Niles said with regard to the fighting propensities of epileptics that he know of a boy who was backward in school but who used frequently to fly at his teacher with great ferocity and who claimed afterwards that he did not know about these attacks. A little later in life he began to have regular convulsive seizures and showed that his previous ill temper was merely preliminary to the epileptic life.

Dr. Brawner said that Dr. Block was correct in stating that a large number of convulsive attacks was what determined the mental degeneration.

---

### REGULAR MEETING FULTON COUNTY MEDICAL SOCIETY, MARCH 16, 1911.

Dr. Wesley Taylor read a paper on "Intoxication Insanities."

Dr Kime said that dietary toxemias are of great importance and are constantly receiving more accurate attention. Vegetarianism, while not advisable as a universal diet, relieves many such cases by eliminating the albuminous putrefaction. The effect on the ductless glands is being studied in this connection and promises great results.

Dr Block related a case of a child who drank one-half pint of whiskey and had alcoholic convulsions several times during two weeks.

Dr. Daly said that the medical legal aspects of these mental conditions were of the utmost importance in that people frequently forgot entirely what they were doing during long periods of time, whether they were subsequently insane or not. This is evidenced by the loss of memory in acute sprees which frequently comes to our attention and also in the amnesias of chronic alcoholism, whether there is fever or not. In talking on this subjects, he added that Georgia stood sadly in need of better provisions for examination of the insane than it has under its present regulations. The incarceration of a sick person in a jail is not a good preliminary to rational treatment. He showed that it was practical to commit patients to the asylum within a few hours and still protect their rights thoroughly against improper detention. A jury trial is unnecessary in the great majority of cases if suitable experts without bias are appointed by the courts to examine the ailing patients.

Dr. Westmoreland said that several years ago in a jail he inspected there were 134 people awaiting examination and some of these had been there from one to three years. The ten-day detention period should be waived in proper cases; it is only an arbitrary rule.

Dr. W. A. Jackson read a paper on "Commercial Value of Sanitary Measures."

Dr. Lindorme said that the chert roads where autos pass are always dusty and dangerous to the householders along the routes and that money might well be spent by the community in providing hygienic surfacing. He called attention to the fact that the Jamestown Exposition was located in a most unhealthful place, but the santary inspection of the army cleaned it up and kept the soldiers healthy when other people no tobeying the regulations and living outside the reservaton suffered disease. At Chicamauga Park the recent maneuvers showed how much better men can live there under sanitary measures than they did during the Spanish-American war. Refuse, etc., are now burned instead of being buried as they were then. The ground is not polluted and there is nothing wrong with the men. No well-water is used without boiling.

Dr. Jackson, in closing, spoke of the difficulties in making healthy men protect themselves as they seem to take it as a joke.

Dr. G. K. Varden read a paper on "Syphilis in Infancy and Early Life."

Dr. Paullin said he recently gave the Wasserman test to a negro child six days old. The symptomatology was similar to that mentioned in the paper; the reaction was positive, as was also the case with the mother. Salvarsan will be given to the mother. One would not expect a positive Wassermann unless anti-bodies were transferred from the mother to the child because the child would not have had time to make them. It is a question whether they can be found during foetal life.

Dr. Westmoreland called attention to Colles law which says that non-syphilitic women can have a syphilitic child, which seemed to antagonize Dr. Paullin's idea.

Dr. Ballenger said that, in his opinion, the mother could not have a syphilitic child unless she, herself, was a syphilitic, but that her symptoms and the action of the disease may be so obscure as to evade all but the most careful observations. He agrees that very small doses of salvarsan should be given to the child. Many babies were killed before they found what the dose was. Most of the organism should be killed in preliminary mercurial treatment.

Dr. Paullin said that the question of Colles law is most interesting. He would like to get more cases of syphilitic children upon which to make the Wassermann test upon both mother and child. He would like the profession generally to send him such cases for scientific investigation at the State Laboratory.

Dr. Archibald Smith said he has a colored woman patient with no apparent symptoms, but the baby two months old has typical lesions. He will send it to Dr. Paullin.

Dr. Block said it was most important to investigate the nervous symptoms of syphilis in childhood, many of which are overlooked. Convulsions and hemiplegias occur and may be called epileptic when they are really luetic.

Dr. Westmoreand told of nine boys in a family, all of whom had syphilis appearing at puberty, but the mother never had any signs of the disease.

Dr. Barnett said that a short time ago he delivered an eclampsia child at 8 1-2 months, covered with specific eruptions. He believes that if the anti-bodies do pass from mother to child that will change our theories of the foetal nutrition.

Dr. Varden said, with regard to salvarsan that he had three cases from 9 to 14 months of age. In small doses two cases did well, but in the third the case was bad at the end of six months, showing loss of power in the lower limbs.

### THE MEDICAL PROFESSION MUST CHANGE ITS TACTICS.

By William J. Robinson, M. D., New York.
*President of the American Society of Medical Sociology.*

He who is not a frequent visitor to radical clubs, does not come in contact with newspaper men, with New Thoughters, and does not read regularly the numerous naturopathic, health culture and physical culture journals, and other allegedly advanced publications, can have no idea how the medical profession is ridiculed, how it is maligned, how it is lied about, how it is misrepresented, how it is "knocked" on every possible occasion.

We are pictured as ignoramuses, grafters, butchers, anxious to operate whether there is a necessity or not, drug dopers, etc., etc. We are denounced as a trust, monopoly and any attempt of ours to organize, to pass laws protecting the public health is characterized as an attempt at class legislation, a desire for special privileges, inspired by our fear of the competition, by our fear of the superior skill of our irregular rivals.

And the average physician who has not given the matter any thought, has no idea what effect these unceasing slanders and persistent lies have on the public mind, how suspiciously a large part of the public is beginning to look at the medical profession, how we are losing the confidence of the people, how the ground is slipping from under our feet.

As an illustration, we need only mention the reception that has been accorded to the suggestion of a Federal Department of Health. The motives that actuate us and the objects of such a department were at once misrepresented, the people were made to believe that their freedom to choose a medical adviser was threatened, the forces of reaction and obscurantism, masquerading in some instances under the guise of liberalism, were quickly marshaled and in a short time a society was organized, which now claims a membership of one hundred and fifty thousand.

We physicians are ourselves to blame. When the irregular, fantastic and pernicious cults began to make their appearance, we paid no attention to them. We thought they amounted to nothing.

and would soon dry up and shrivel away of themselves. When the malicious attacks began to appear in the various quack publications, we remained silent. We considered it *infra dignitatem* to pay attention to them, and we thought that the public would have no difficulty in seeing through their falsity and meretriciousness.

Our long and patient inactivity has been due to the false idea that truth will always triumph and error is bound to die. Yes, eventually. But if error is allowed to grow and spread unhampered, while those who see the truth will not take the trouble to proclaim it and expose the error, then it can take centuries before the correctness of the truth and the falsity of the error will be perceived.

In this as in every line of human activity prevention is immeasurably superior to cure, and the right way to fight error is not to permit it to get a firm foothold. Error and superstition are hard things to uproot after they have attained the dignity of a universal belief.

It is time that the medical profession change its tactics and assume a wideawake, militant attitude. It is time that we actively attack error wherever it shows its head. By reading papers before lay audiences, by participating in discussions, by writing to the newspapers, by refuting the false arguments of the false prophets wherever they appear, we can do much toward destroying the influence of the quacks and the irregular cults. In short, we must throw off our exclusiveness, we must go out to the public and take it into our confidence.

The truth is with us—that we know, only we must not hide it under a bushel and expect that its light will, wthout any effort on our part, penetrate into the darkest recesses of ignorance and quackery.

---

### THE STATE TUBERCULOSIS SANATORIUM.

The state tuberculosis sanatorium at Alto, in Banks county, has been formally opened, and already seven patients have availed themselves of the opportunity for treatment. The capacity of the institution is 70 patients, and it is believed that the limit will be reached soon, once the people throughout the state realize that it is open and appreciate the great work that has been undertaken.

Capt. William G. Raoul, chairman of the executive committee, states that the capacity of the sanatorium may be increased at the pleasure of the legislature at comparatively little expense. The buildings have been so constructed that room for 300 patients may be easily provided.

"Admission to the tuberculosis sanatorium will be made upon the recommendation of examining physicians," said Captain Raoul. "Patients desirous of entering the Alto institution will be admitted in the order of their applications, provided, of course, the applications bear the indorsement and recommendation of a physician. The sanatorium is open to the white citizens of Georgia, who have been residents of the state at least one year preceding the date of their application for admittance, and whose physical condition justifies hope of recovery. The present equipment and organization is not yet adapted to the treatment of advanced cases of tuberculosis, and until these improvements have been made no such patients will be received."

---

### A LETTER TO A DOCTOR WHO IS ABOUT TO ENTER THE ROAD WHICH LEADS TO QUACKERY.

By William J. Robinson, M.D., New York.

*President of The American Society of Medical Sociology.*

*Dear Doctor*:—I do not know you. I have never met you. And still I am going to write to you. I do so as a matter of duty. To the question: Am I my brother's keeper? I have always answered: Yes. And I see a young man starting on a wrong path, I try to warn him, to save him, if I can.

You have entered upon the wrong path, and I am sorry for you. And I urge you to turn back while there is yet time. I advise you to turn from the path of quackery, upon which you are, consciously or unconsciously, entering. I advise you to do so, not only moral and ethical reasons, but because you yourself will in the end be the greatest sufferer. The man who never graduated from a medical college, the man who was never associated with the medical profession, the man who is an out-and-out quack, has no such scruples as you will be tortured by; he cares only for the money, and as long as he makes that he is satisfied. But you will suffer. When you see your professional colleagues turning away from you, when you see yourself avoided by even your personal medical friends, when you see yourself shut out from all medical societies, you will suffer. You may put up a bold front, you may try to make others and yourself believe that you do not care, that you do not give a rap for the approbation of your medical brethren, that their ostracism is nothing to you. But deep down in your soul you will feel that it is not so; you will feel that you are deceiving yourself as well as others; you will pass many sleepless nights, and your pillow will hear some heart-breaking confessions.

How do I know it? I know it because many of the better class of quacks have made confessions to me. One man, who has made a pile of money and who, in spite of his undoubtedly quackish methods, has many excellent qualities and humanitarian inclinations, confessed to me that he would willingly exchange the plaudits of fifty thousand laymen for the approbation of one reputable medical man.

Of course, I know what you will say. You will say that you are knocking the medical profession, and going to the laity, because the medical profession is narrow, bigoted, commercialized, and so forth. I am even willing to believe that that is your real reason for leaving the profession, and not the fact that in the ordinary ethical practice of your profession you were unable to make a satisfactory living. But, still I would ask you to ask yourself this question: Would I have left the path of regular ethical medicine if in following it I had been making five or ten thousand dollars a year? I fear your answer would be in the negative.

But, assuming that it is so, that the profession is narrow, commercialized, etc., is it not your duty to stay on the inside and fight this narrowness and commercialism, and other abuses which have crept in the profession? Don't tell me that you cannot do it and still remain within the profession. For it is not so. I myself am the proof of it. Nobody has on occasions criticized the shortcomings of our profession more severely than I have. THE CRITIC AND GUIDE was organized for the distinct purpose of criticizing the abuses and faults of the medical and pharmaceutical professions. But while some narrow bigots did not like my criticizing, this did not put me out of the pale of the profession. I stayed right in, fought as hard as I knew how, and with the result that it was I, and not the bigots, that carried off the victory. It is not I that became narrower, it is the bigots who became broader—or retired into solitude.

Don't you see that by leaving the profession—for the path you are entering upon means essentially this—you render yourself entirely impotent for any good? Even the laity will cease to listen to you as soon as they find out that by the regular profession you are considered a quack. And what's more, when they want a doctor, they will consult a regular physician. Finding yourself forsaken by the profession, and by the laity, you may want to turn back to the former, but you may find the portals shut. For our profession is a jealous profession and is not lenient towards transgressors.

The medical profession, that is, a part of it, for to accuse the entire profession would be wrong, may be narrow and commercialized. No profession can help being affected by the tendencies of our times, by the influence of our social and economic conditions. But a profession that counts among its members a Hippocrates, a Galen, a Harvey, a Servetus, a Vesalius, a Pare, a Maimonides, a Louis, a Troasseau, a Fournier, a Graefe, a Virchow, a Muller, a Von Helmholz, a Gerhardt, a Billroth, an Ehrlich, an Osler and a Jacobi, is a pretty good profession to belong to.

Don't you think so?

Or do you perhaps think that the company of old "Dr." Grindle and "Dr." Gray and "Dr." Tilden and "Prof." Samuels and Mr. McFadden is preferable? If so, I have nothing to say.

*Chacun a son gout.*

Still fraternally yours,

WILLIAM J. ROBINSON.

